# Post-infectious Transverse Myelitis Secondary to Hand, Foot, and Mouth Disease in a Pregnant Daycare Worker

**DOI:** 10.7759/cureus.56159

**Published:** 2024-03-14

**Authors:** Mohammad Jahangiri, Akhil Padarti, William A Kilgo

**Affiliations:** 1 Neurology, University of South Alabama, Mobile, USA

**Keywords:** enterovirus, magnetic resonance imaging, hand foot & mouth disease, transverse myelitis, central nervous system inflammation

## Abstract

Transverse myelitis (TM) is a rare inflammatory disorder of the spinal cord that infections, vaccines, and autoimmune processes can cause or may have no discernible cause. About half of the cases are caused by an infection, usually a viral respiratory infection, flu-like illness, or sometimes a gastrointestinal infection. Although coxsackieviruses and enteroviruses are known to cause TM, it is more commonly associated with respiratory symptoms or systemic signs than a rash. In this case, we present a pregnant daycare worker who had a case of longitudinally extensive TM after an episode of hand, foot, and mouth disease (HFMD), which only showed the typical rash without fever or systemic signs.

## Introduction

Transverse myelitis (TM) is a rare, focal inflammatory disorder of the spinal cord that typically presents with acute to subacute development of motor, sensory, and autonomic (bowel, bladder, and sexual) dysfunction up to the affected level of the spinal cord. Incidence is approximately one to five cases per million per year, excluding acquired demyelinating diseases such as multiple sclerosis (MS). It is most often postinfectious or postvaccination but can also occur in the setting of systemic autoimmune disorders (e.g., systemic lupus erythematosus [SLE]) or primary autoimmune disorders of the central nervous system, for example, neuromyelitis optica spectrum disorder (NMOSD) and MS. There are also rare etiologies, including paraneoplastic and drug/toxin-induced [1]. If MS is included, the incidence increases to approximately 25 cases per million [2]. Approximately half of the non-MS cases are postinfectious. In one study involving 33 patients (aged 18 months to 82 years), 46% had a preceding infection of which 73% were respiratory, 13% were gastrointestinal, and 13% had flu-like symptoms [3,4].

Hand, foot, and mouth disease (HFMD) is an infectious disease caused by enteroviruses, most often enterovirus 71 (A71) and coxsackievirus A16, characterized by a papulovesicular rash affecting the palms, soles, and buttocks accompanied by fever and stomatitis. It typically affects children under the age of 10, as well as occasional immunocompetent adult contacts, through a fecal-oral route of transmission [5]. Here, we present a case of longitudinally extensive TM preceded by an episode of HFMD in a pregnant daycare worker without systemic signs or fever. To our knowledge, there is only one other case report describing a similar occurrence of TM after HFMD [6].

## Case presentation

Our patient was a 25-year-old pregnant female in the second trimester of gestation. Approximately two weeks before the onset of symptoms, there was an outbreak of HFMD at the daycare facility where she worked. Her symptoms started with a vesicular rash on the palms of her hands and soles of her feet. At that time, there were no other systemic symptoms such as fever or myalgias. This rash resolved quickly over two to three days. Neither the children at daycare nor the affected staff underwent testing to identify the causative viral agent of HFMD. Until the onset of the rash, the patient was following up with her obstetrician regularly and had no complications with her pregnancy.

**Figure 1:**
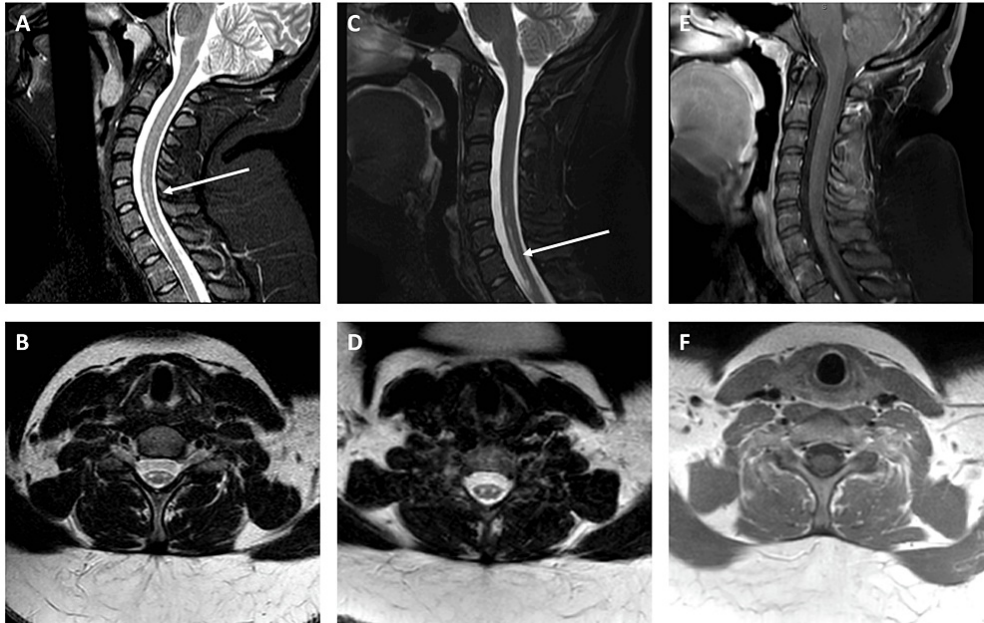
Initial and repeat magnetic resonance imaging (MRI). MRI during the initial hospitalization and repeat imaging. (A) MRI cervical spine (C-spine) sagittal T2 STIR imaging taken at the time of hospitalization shows signal changes from C5 to T1. The white arrow highlights the cord signal changes. (B) MRI C-spine axial T2 turbo spin-echo (TSE) imaging, taken at the time of hospitalization around the C7 vertebral body, reveals signal changes in the anterior columns. (C) MRI C-spine sagittal T2 STIR imaging taken seven months after the episode shows signal changes from C5 to T1. The white arrow highlights the cord signal changes. (D) MRI C-spine axial T2 TSE imaging taken seven months after the episode at approximately C7 vertebral body shows signal changes at the anterior columns. (E) MRI C-spine sagittal T1 post-contrast imaging taken seven months after the episode does not show any contrast enhancement within the spinal cord. (F) MRI C-spine axial T1 post-contrast imaging taken seven months after the episode at approximately C7 vertebral body does not show any contrast enhancement within the spinal cord.

Two weeks after the initial rash, she began to develop severe sharp, tight pain around her shoulder blades with radicular pain radiating down her arms. Soon after the development of her paresthesia, she went to the emergency room and was initially discharged home with a diagnosis of radiculopathy. However, over the next 24 hours, she developed progressive weakness, sensory loss, and urinary retention. She returned to the emergency department. Her initial physical exam at the second emergency department visit showed significant bilateral lower and upper extremity weakness (1-2/5) and numbness. She was no longer able to transfer from the wheelchair to the bed by herself. Due to the rapid progression, she was admitted to the hospital. Magnetic resonance imaging (MRI) at that time revealed an abnormally increased T2/STIR signal in the bilateral anterior columns of the spinal cord from C5-T1 without structural compression, suspected to be acute TM (Figure 1). Unfortunately, no contrast-enhanced studies were available from the outside hospital. She underwent a lumbar puncture, and cerebrospinal fluid (CSF) results were unremarkable (Table 1). Broad laboratory workup evaluating for other causes of TM was obtained and deemed negative (Table 2). The patient underwent treatment with high-dose steroids (1 g of methylprednisolone daily) for five days. Strength in her arms and legs improved over that period.

**Table 1:** Cerebrospinal fluid testing. All relevant testing was conducted on the cerebrospinal fluid. The second column represents the normal range for the studies, while the third column displays the patient results for the studies. WBCs, white blood cells; RBCs, red blood cells; IgG,  immunoglobulin G; ACE, angiotensin-converting enzyme; CSF, cerebrospinal fluid; PCR, polymerase chain reaction; CMV, cytomegalovirus; VZV, varicella-zoster virus; HSV, herpes simplex virus; HHV, human herpes virus

	Normal value	Patient value
WBCs	0-5 cells/mcL	0
RBCs	0-2 cells/mcL	0
Glucose	40-70 mg/dL	56
Protein	15-45 mg/dL	25
Oligo clonal bands	0 bands	0
IgG index	0.32-0.60 U	0.5
ACE Level	0-2.5 U/L	0.5
CSF PCR	Undetectable for CMV, VZV, HSV-1, HSV-2, HHV-6, Enterovirus, Cryptococcus, Haemophilus influenzae, Listeria monocytogenes, Neisseria meningitidis, Streptococcus agalactiae, and Streptococcus pneumoniae	

**Table 2:** Serum laboratory testing. All relevant testing was conducted on the serum. The second column represents the normal range for the studies, while the third column displays the patient results for the studies. Abnormal values are bolded. SSA, Sjogren syndrome-related antigen A autoantibodies; SSB, Sjogren syndrome-related antigen B autoantibodies; IgG, immunoglobulin G; NMO, neuromyelitis optica; AQP4, anti-aquaporin 4 antibody; MOG, myelin oligodendrocyte glycoprotein; HgA1C, hemoglobin A1C; RPR, rapid plasma reagin; HIV, human immunodeficiency virus

	Normal value	Patient value
SSA (Ro) antibody, IgG	<5 U/L	2 U/L
SSB (La) antibody, IgG	<5 U/L	0
Myeloperoxidase, antibodies, IgG	<5 U/L	1
Serine protease 3, IgG	<5 U/L	1
ANA	Negative	Negative
Rheumatoid factor	<15 U/L	<15 U/L
NMO/AQP4	Negative	Negative
MOG	Negative	Negative
Copper	62-140 mcg/dL	232.5 mcg/dL
Ceruloplasmin	20-40 mg/dL	49 mg/dL
Vitamin B12 level	190-950 pg/mL	350 pg/mL
HgA1C	<5.0 U	<5.0
RPR	Nonreactive	Nonreactive
HIV Ab	Nonreactive	Nonreactive

After discharge, she was referred to our neuroimmunology clinic from an outside hospital to evaluate the etiology of an episode of TM. Her repeat physical exam showed overall improvement, but it was still notable for diffuse spasticity and weakness in the left hand (4/5 strength in all major muscle groups except 3/5 strength in the left wrist flexors, left finger flexors/extensors, and intrinsic muscles of the left hand). The sensory exam was notable for a deficit up to the T3 dermatome to light touch, pinprick, and temperature. After physical therapy for several weeks, she was able to walk on her own but occasionally required assistive devices over longer distances due to recurrent falls. Her pregnancy continued without any other complications. The patient was continually followed in the neuroimmunology clinic for the next three years. She eventually regained use of her left hand but continued to complain of it locking up and cramping periodically. She never had a relapse or new neurological symptoms after her initial episode consistent with a monophasic event.

## Discussion

TM is a known complication of enterovirus infections and is frequently seen in children [7]. However, the following few features of our patient’s history are unique:

(1) The patient was an adult with a longitudinally extensive TM after a primary enterovirus infection.

(2) TM was not preceded by any systemic signs such as fever, myalgias, or other infectious symptoms as one might expect from a viral illness.

The Transverse Myelitis Consortium Working Group has well-established guidelines for the diagnosis of (idiopathic) TM [8]. Our patient met clinical criteria, with the possibility of structural causes ruled out by imaging. However, no gadolinium-enhanced MRI cervical spine imaging was available for our review during the initial hospitalization, and follow-up images over the years showed no persisting enhancement (Figure 1). In our case, the serum and CSF laboratory workup revealed no significant findings (Tables 1-2). The patient had an acute presentation rather than subacute or chronic myelopathy and had clinical improvement with steroids, suggestive of an infectious/inflammatory etiology. Other causes of TM such as myelin oligodendrocyte glycoprotein-associated disease (MOGAD), NMO, sarcoidosis, and other rheumatological disorders were ruled out with screening labs. Therefore, her clinical presentation suggested a postinfectious etiology as the most likely cause.

Enteroviruses, including those that cause HFMD, are well-known for their association with TM, exhibiting a specific affinity for anterior horn cells [9,10]. Our patient’s imaging reflects this distinct finding. MRI showed T2/FLAIR change in the anterior column adjacent to the anterior horn cells. PCR of CSF samples is not a sensitive way to detect enterovirus infections with neurological complications despite evidence of direct viral invasion of anterior horn cells in studies using mouse models, which was also supported by studies documenting regional outbreaks [10-12]. For example, during a 2018 outbreak of enterovirus A71 in Colorado, enterovirus was only detected using RT-PCR in 20% of CSF samples as opposed to 94% of rectal and 79% of oropharyngeal samples [13]. This was consistent with its known fecal-oral mode of transmission. For these reasons, CSF testing did not detect any enterovirus in our case [14,15]. In our case, the rectal and oropharyngeal samples were not obtained for viral PCR testing.

We searched the literature for similar case reports and case series on PubMed and Google Scholar using a combination of the terms “hand foot mouth disease,” transverse myelitis,” “adult,” and “enterovirus” and discovered only one other case report of an adult patient developing longitudinally extensive TM after infection with HFMD [6]. Unlike our case, this patient presented with febrile illness. On the other hand, TM is a rare but known complication of HFMD reported in children.

## Conclusions

To our knowledge, there is only one other case report involving another adult patient who developed longitudinally extensive TM after infection with HFMD. This is also the first case with mild symptoms of a rash, without accompanying systemic signs or symptoms. Despite no laboratory evidence of enteroviral symptoms, no contrast-enhanced MRI of the cervical spine, and no CSF pleocytosis, it is possible to narrow down the causative factor by history and imaging findings alone as was in our case. This case highlights the possibility that patients can present with postinfectious TM where infectious symptoms resolve before neurological symptoms arise and can be due to uncommon pathogens.
